# Potential Influence of *NUCB2* Genetic Variants on the Clinicopathological Features of Patients with Hepatocellular Carcinoma

**DOI:** 10.3390/diagnostics16203400

**Published:** 2026-10-19

**Authors:** Sung-Lin Hu, Hsiang-Lin Lee, Ming-Yu Lien, Edie-Rosmin Wu, Xiu-Yuan He, Shun-Fa Yang, Chih-Hsin Tang

**Affiliations:** 1 School of Medicine, China Medical University, Taichung 404333, Taiwan; 013437@tool.caaumed.org.tw (S.-L.H.); leinmirain@hotmail.com (M.-Y.L.); 2 Department of Family Medicine, China Medical University Hsinchu Hospital, Hsinchu 302044, Taiwan; 3 School of Medicine, Chung Shan Medical University, Taichung 40201, Taiwan; s31079@gmail.com; 4 Department of Surgery, Chung Shan Medical University Hospital, Taichung 40201, Taiwan; 5 Division of Hematology and Oncology, Department of Internal Medicine, China Medical University Hospital, Taichung 404327, Taiwan; 6 Division of General Surgery, Changhua Christian Hospital, Changhua 500209, Taiwan; desellyn540@gmail.com; 7 Department of Pharmacology, School of Medicine, China Medical University, Taichung 404333, Taiwan; hchp99812@gmail.com; 8 Institute of Medicine, Chung Shan Medical University, Taichung 40201, Taiwan; 9 Department of Medical Research, Chung Shan Medical University Hospital, Taichung 40201, Taiwan; 10 Office of Research and Development, Asia University, Taichung 413305, Taiwan; 11 Chinese Medicine Research Center, China Medical University, Taichung 40447, Taiwan

**Keywords:** nesfatin-1, *NUCB2*, hepatocellular carcinoma, genetic polymorphisms, smoking, vascular invasion

## Abstract

**Background:** Hepatocellular carcinoma (HCC) is the most commonly diagnosed cancer type worldwide in males and the ninth among females, and it is a primary cause of cancer-associated death. The adipokine nesfatin-1, initially discovered in hypothalamic neurons, is produced by the precursor *NUCB2* gene. The relationship between *NUCB2* polymorphisms, behavioral risk factors that facilitate cancer, and HCC is still unknown. **Methods:** Four *NUCB2* genetic polymorphisms were investigated in 822 normal controls and 411 patients with HCC using qPCR. The GTEx portal was used to assess the association between *NUCB2* genetic variants and expression. NUCB2 levels in patients with HCC were evaluated using TCGA data. **Results:** Relative to the wild-type GG genotype, the AA genotype at the *NUCB2* SNP rs214101 was associated with an augmented risk of distant metastasis and vascular invasion. The GA and AA genotypes at rs214101 were also associated with an increased likelihood of vascular invasion among smoking HCC patients compared to those carrying the GG genotype. Notably, GTEx data uncovered that the AA homozygous genotype was correlated with remarkably higher NUCB2 levels than those observed in individuals with the rs214101 GG genotype in lymphocytes and whole blood samples. TCGA data revealed that HCC patients exhibited higher NUCB2 expression in tumorous compared to non-tumorous liver tissue. **Conclusions:** *NUCB2* SNP rs214101 may be associated with HCC progression and metastasis.

## 1. Introduction

Hepatocellular carcinoma (HCC) is the most commonly diagnosed cancer type worldwide in males and the ninth among females, and it is a primary cause of cancer-associated death [1]. HCC is associated with rising death rates and a poor five-year survival rate [2,3]. HCC is the second-highest contributor to deaths attributable to cancer in Taiwan [4,5]. HCC susceptibility and development depend heavily on genetic variation. The majority of people with known environmental or viral risk factors (especially alcohol abuse, obesity, viral hepatitis infection, diabetes or non-alcoholic fatty liver diseases associated with insulin resistance) fail to develop HCC. This suggests that carcinogenesis is influenced by individual susceptibility [6,7]. Recent studies have highlighted the importance of identifying prognostic biomarkers and therapeutic targets for HCC. Qian et al. identified and validated tanshinone IIA-related prognostic genes in HCC, highlighting their potential value in predicting patient outcomes and guiding therapeutic strategies [8]. In patients with HCC, metastasis is a major contributor to cancer-related mortality. Tumor cells can spread through vascular invasion, particularly via the portal and hepatic veins. Tumor microenvironment remodeling, angiogenesis, and epithelial–mesenchymal transition are important processes associated with HCC metastasis, contributing to poor clinical outcomes and limited treatment options [9,10].

An increasing amount of evidence points to a link between obesity and cancer [11,12]. Colorectal, pancreatic, and prostate cancers are among the tumors where this connection is most noticeable [11,13]. Moreover, obesity can change the microenvironment of cancer, hastening its development [14]. Adipose tissue secretes bioactive substances called adipokines, which control many pathogenic processes. Adipokines affect differentiation, apoptosis, proliferation, angiogenesis, invasion, and metastasis, among other aspects of cancer [15,16]. Nesfatin-1 was first discovered in neurons in the hypothalamus and is derived from its precursor *NUCB2* [17]. Expression of nesfatin-1 has also been observed in several peripheral organs, including fat tissue, the pancreas, and the ovaries [18]. In addition to its well-established role in the regulation of appetite and energy homeostasis, nesfatin-1 has been reported to exert anti-inflammatory and antioxidant effects [19,20]. Increasing evidence also suggests that the NUCB2/nesfatin-1 system may be involved in the development and progression of malignant diseases. Increased expression of NUCB2 has been observed in several types of malignancies, including thyroid, prostate, colorectal, endometrial, and breast cancers [21]. In these malignancies, dysregulation of NUCB2/nesfatin-1 has been associated with tumor-related biological behaviors, such as cell proliferation, migration, and invasion, and may be related to unfavorable clinical outcomes and reduced recurrence-free survival. These findings suggest that NUCB2/nesfatin-1 may play a potential role in tumor progression and may serve as a molecular link between metabolic regulation and malignant behavior.

A single nucleotide at a specific position in the genome is altered in a single-nucleotide polymorphism (SNP), the most common type of genetic variation. SNPs can affect biological processes, gene expression, and a person’s vulnerability to a number of illnesses [22]. To evaluate the clinical outcome and risk of illnesses, such as cancer, comparison of SNP frequencies between patient cohorts are frequently conducted [23]. The connections between carcinogenic behavioral variables, *NUCB2* genetic polymorphisms, and HCC are unknown. Therefore, this study looked at the effects of carcinogenic behavioral mediators and *NUCB2* gene polymorphisms on the risk of developing HCC among Taiwanese individuals.

## 2. Materials and Methods

### 2.1. Study Participants

This study included a total of 411 patients with HCC registered at Chung Shan Medical University Hospital, Taiwan. All participants were of Han Chinese ethnicity. The demographic and clinical characteristics of the study participants were collected from the available clinical records and the Taiwan Biobank database. The 2010 American Joint Committee on Cancer (AJCC) TNM staging approach, which considers tumor form and the number of metastases and afflicted lymph nodes, was used to stage patients with HCC [24]. Each participant finished a standardized questionnaire assessing sociodemographic characteristics and alcohol and cigarette usage, and provided informed written consent prior to enrollment in this project. Liver cirrhosis was diagnosed based on the biopsy findings, appropriate sagittal MRI or CT images, or biochemical markers of liver parenchymal destruction combined with endoscopic gastric or esophageal varices. This investigation was approved by the Institutional Review Board of Chung Shan Medical University Hospital prior to its initiation (IRB No. CS2-19133).

### 2.2. Selection and Genotyping of SNPs

The *NUCB2* SNPs rs10766383, rs214101, rs1330, and rs757081 were chosen based on earlier publications [25,26]. According to the dbSNP database, the minor allele frequencies (MAFs) of rs10766383 (T>G), rs214101 (G>A), s1330 (C>T), and rs757081 (C>G) in the Asian population are 0.495 (G), 0.191 (A), 0.361 (T), and 0.360 (G), respectively. Genomic DNA was extracted from 3 mL peripheral blood samples using the QIAamp DNA Blood Kit (QIAGEN, Hilden, Germany). NUCB2 SNPs were subsequently analyzed using TaqMan-based SNP genotyping assays with allelic discrimination [27], and the genotyping data were analyzed using SDS 7000 series software, version 1.2. (Applied Biosystems, Foster City, CA, USA).

### 2.3. Analysis of Clinical Dataset

A thorough open-access resource for evaluating tissue-dependent gene expression and regulation is the GTEx portal. It offers histological images and publicly available gene expression and quantitative trait locus (QTL) data [28]. We conducted an additional study to select HCC patients from The Cancer Genome Atlas (TCGA). NUCB2 mRNA expression data for HCC were obtained from The Cancer Genome Atlas (TCGA) liver hepatocellular carcinoma (LIHC) dataset. The analysis included 371 HCC tumor samples and 50 normal liver tissue samples. For paired analysis, 50 matched tumor–normal tissue pairs were evaluated. NUCB2 expression was analyzed using the RSEM expression values available from the TCGA dataset.

### 2.4. Statistical Analysis

The HCC and control groups were compared using the Mann–Whitney U test and Fisher’s exact test; *p* < 0.05 was considered statistically significant. The odds ratios (ORs) and matching 95% confidence intervals (CIs) for the relationships between genotype frequencies and HCC risk were estimated utilizing logistic regression analysis. The adjusted odds ratio (AOR) was adjusted for age, gender, cigarette smoking, and alcohol consumption. Differences in NUCB2 expression between unpaired tumor and non-tumorous liver tissues were evaluated using the Wilcoxon rank-sum test, whereas differences between paired tumor and non-tumorous liver tissues were assessed using the Wilcoxon signed-rank test. Statistical Analysis System (SAS) software, version 9.1, was applied to statistically assess all of the data that was gathered.

## 3. Results

There were no discernible differences in the demographics of the 822 cancer-free controls and the 411 patients with HCC (Table 1). The proportions of cigarette smokers in the two groups were identical (*p* = 0.456), but the controls reported drinking alcohol much less frequently than patients (*p* < 0.001) (Table 1). The percentages of HCC patients who tested positive for anti-HCV antibodies and HBsAg were 36.5% and 45% (Table 1). A total of 309 patients (75.2%) had stage I/II HCC at the time of study enrollment, whereas 102 patients (24.8%) had stage III/IV illness. Additionally, patients diagnosed with HCC showed vascular invasion (12.2%), metastasis (5.8%), and N1+N2+N3 lymph node status (2.2%). The majority of patients (86.4%) had liver cirrhosis (Table 1).

The genotyping information for the *NUCB2* SNPs in HCC patients and normal controls is presented in Table 2. The genotype distributions of rs1330, rs757081, and rs10766383 in the control group were consistent with Hardy–Weinberg equilibrium (HWE) (χ^2^ = 0.978, *p* = 0.323; χ^2^ = 2.354, *p* = 0.125; and χ^2^ = 0.013, *p* = 0.910, respectively). In contrast, the genotype distribution of rs214101 showed a slight deviation from HWE (χ^2^ = 3.999, *p* = 0.046). The most common genotypes were homozygous C/C for rs1330, homozygous G/G for rs214101, homozygous C/C for rs757081, and homozygous T/T for rs10766383. After adjusting for age, gender, alcohol use, and cigarette smoking, no genotype among the four *NUCB2* SNPs in the comparator groups demonstrated a notable relationship with HCC (Table 2).

Subsequently, an evaluation of differences in clinical characteristic between different *NUCB2* genotypes in HCC patients was performed. The clinicopathologic features of HCC patients were not significantly impacted by SNPs rs1330, rs757081, and rs10766383. However, patients carrying the AA genotype at rs214101 exhibited an elevated risk of distant metastasis (OR = 4.078; 95% CI: 1.044–15.923; *p* < 0.05) and vascular invasion (OR = 4.276; 95% CI: 1.367–13.374; *p* < 0.05) compared with those carrying the GG genotype (Table 3). Crucially, carriers of the GA or AA genotype at rs214101 correlated with an augmented risk of vascular invasion (OR = 2.818; 95% CI: 1.141–6.960; *p* < 0.05) in patients who smoke, compared with the GG genotype (Table 4).

**Figure 1:**
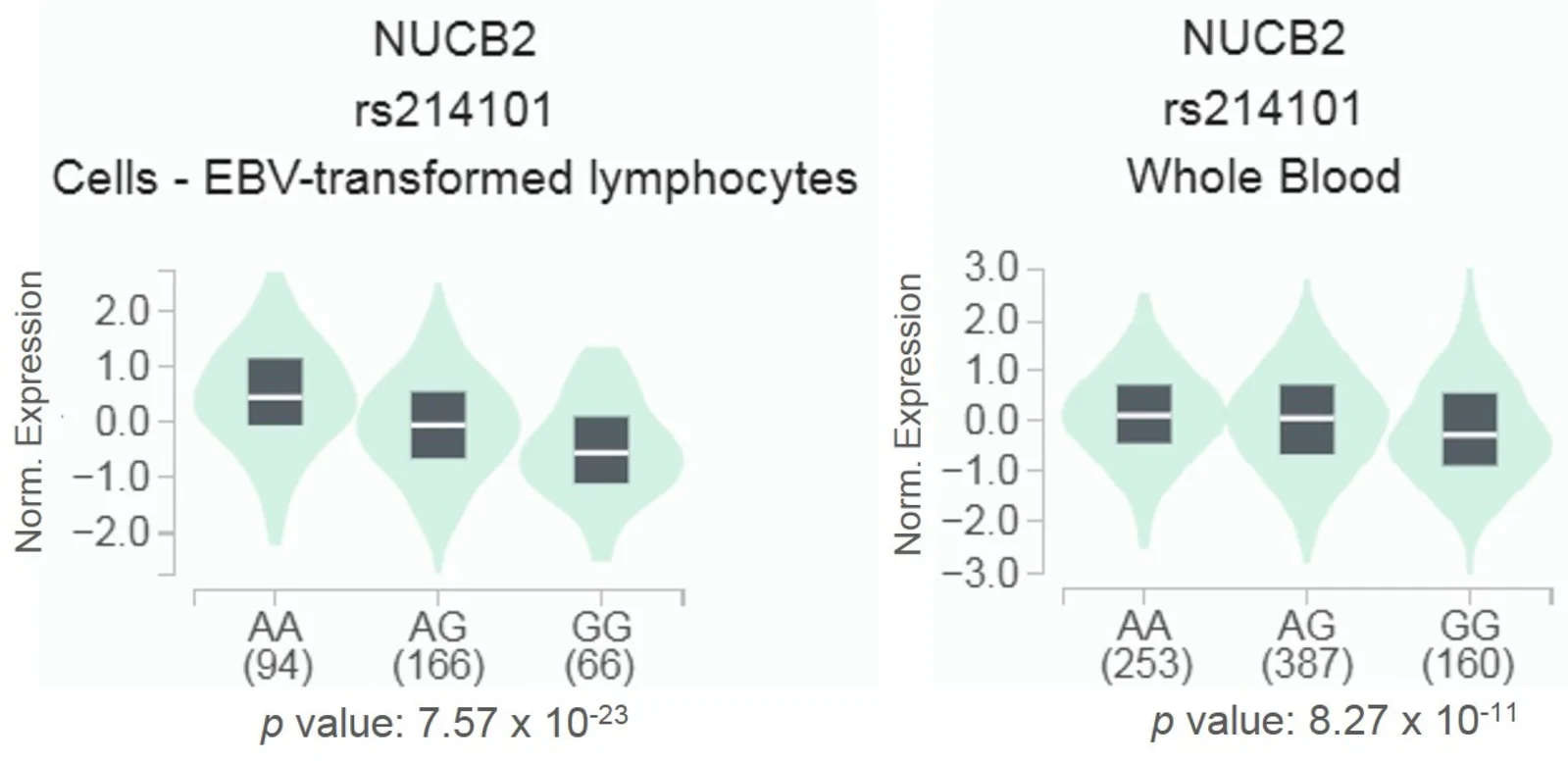
NUCB2 shows a substantial expression quantitative trait locus (eQTL) association with rs214101 genotypes in lymphocytes and whole blood samples from the GTEx database.

**Figure 2:**
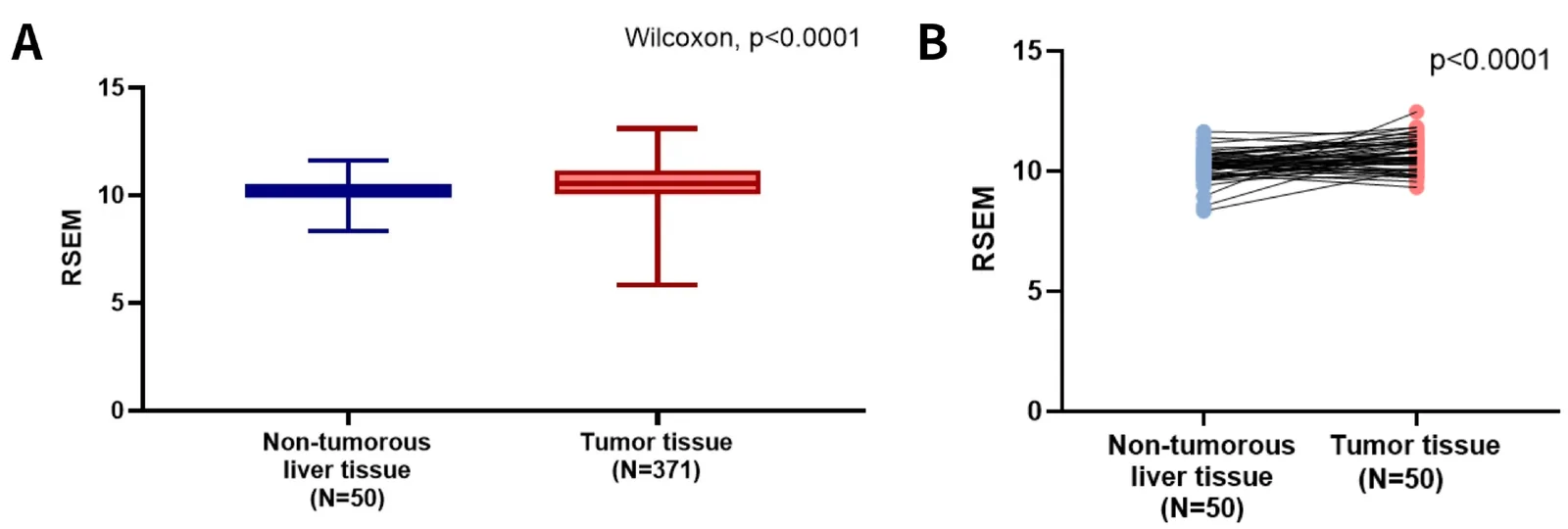
The NUCB2 mRNA levels of HCC patients from TCGA database. (**A**) The NUCB2 levels are increased in HCC patient tissues (red) compared to non-tumorous liver tissues (blue) from TCGA database. (**B**) The paired dot plot indicates that HCC expression was higher in tumor tissues compared to paired non-tumorous liver tissues. RSEM: RNA-Seq by Expectation Maximization.

According to the GTEx data, individuals with the AA homozygous genotype had significantly higher NUCB2 expression levels than those carrying the rs214101 GG genotype in both EBV-transformed lymphocytes and whole blood (*p* < 0.05; Figure 1). In EBV-transformed lymphocytes, the median NUCB2 expression levels were 0.4266, −0.0691, and −0.5430 in the AA, AG, and GG groups, respectively. In whole blood, the corresponding median expression levels were 0.1176, 0.0485, and −0.2660, respectively. Next, we utilized TCGA data to evaluate the relationship between *NUCB2* mRNA levels and tumor stage in HCC patients. Relative to non-tumorous liver tissues or matched normal counterparts, we discovered that tumor tissues had greater *NUCB2* expression (Figure 2).

## 4. Discussion

Numerous tumor studies have shown the value of biomarkers of genetic anomalies linked to cancers in determining risk, facilitating early detection, and predicting therapy outcomes [29,30]. About 1% of people have individual variations or genetic polymorphisms in their genomic sequences. The most prevalent kind of alteration seen in repeated sequences are SNPs [31]. A growing corpus of research has emerged that emphasizes the importance of SNPs and additional genetic variants in determining and estimating therapeutic responses in prostate tumors [32]. The comprehensive characterization of functional variations connected to tumor risk has also indicated a role of SNPs in tumor biology by demonstrating how SNPs in functional areas are associated with tumor susceptibility and gene expression. Oral cancer has been connected to *NUCB2* gene polymorphisms [27]. However, it is still unknown how *NUCB2* polymorphisms relate to HCC. As a result, we looked at *NUCB2* gene polymorphisms and noted how differently they were distributed among HCC patients. Here, we found that the *NUCB2* SNP rs214101 is associated with an increased risk of vascular invasion and distant metastasis, with a more notable association observed for vascular invasion among smokers.

Adipose tissue is strongly linked to the development of HCC [33]. Many investigators have been studying the role of fat tissue in promoting inflammation and tumorigenesis for the past 20 years [34]. Recent research has shown that nesfatin-1 is critical for both cell progression and the induction of tumor cell elimination [21]. Nesfatin-1 levels are higher in breast, thyroid, and colon tumors than in adjacent nonmalignant tissues [35,36]. Moreover, augmented nesfatin-1 levels are strongly associated with advanced-stage tumors and metastasis in renal cell cancer patients [37]. Accordingly, we evaluated differences in genotype and allele frequencies of *NUCB2* genetic polymorphisms between patients with HCC and HCs. The two groups did not differ significantly in total susceptibility to HCC and the four *NUCB2* SNPs. On the other hand, when we categorized HCC patients based on clinical parameters, carriers of the AA genotype at the *NUCB2* SNP rs214101 showed an augmented risk of developing distant metastasis and vascular invasion compared with those carrying the GG genotype. Remarkably, GTEx data indicated that the AA homozygous genotype was associated with markedly elevated *NUCB2* expression relative to the GG genotype of rs214101 in whole blood samples and lymphocytes. Furthermore, TCGA data revealed that HCC patients exhibit higher NUCB2 expression in tumorous than non-tumorous liver tissue. These findings suggest that rs214101 may have a functional effect on NUCB2 expression; however, the GTEx eQTL evidence is derived from whole blood and lymphocytes and should not be directly extrapolated to NUCB2 expression or function in hepatocytes or HCC tissues. Therefore, the observed eQTL association provides supportive evidence for a potential functional consequence of rs214101 but does not establish its tissue-specific biological mechanism in HCC. Further studies using HCC tissues and hepatocyte or HCC cell models are warranted to determine whether rs214101 directly regulates NUCB2 expression and contributes to HCC progression and metastasis.

One known risk factor for HCC is cigarette smoking [38]. Carcinogens found in tobacco smoke include nitrosamines and polycyclic aromatic hydrocarbons, which the liver breaks down into reactive intermediates that can cause oxidative stress, DNA damage, and genetic alterations [39]. These actions encourage a pro-inflammatory hepatic milieu and aid in the development of hepatocarcinogenesis. According to epidemiological research, smokers are more likely to develop HCC, and this risk increases with cumulative exposure [38]. Furthermore, smoking can increase the risk of disease and might lead to more aggressive tumor progression when combined with other predisposing factors, for instance, alcohol use and chronic hepatitis B or C infection [40]. In the present study, among patients with HCC, the GA+AA genotypes of *NUCB2* rs214101, compared with the GG genotype, were associated with an increased risk of vascular invasion in smokers. One possible explanation is that smoking-related oxidative stress, DNA damage, and inflammatory responses may contribute to a tumor-promoting microenvironment, potentially modifying the association between genetic susceptibility and tumor progression. Previous studies have suggested that genetic polymorphisms in carcinogen-metabolizing enzymes, including CYP1A2 and GSTT1, may interact with cigarette smoking to influence the risk of HCC [41,42]. However, whether a similar interaction exists between cigarette smoking and *NUCB2* rs214101 remains unclear and warrants further investigation.

Beyond genetic and clinicopathological biomarkers, advances in precision and molecular imaging may provide complementary approaches for characterizing tumor biology and disease progression. Multimodality imaging can integrate anatomical, functional, and molecular information to improve lesion characterization and assessment of treatment response [43]. Moreover, molecular imaging strategies that distinguish tumor-specific targeting from nonspecific accumulation may provide additional insights into tumor biology and heterogeneity [44]. Integrating molecular biomarkers such as *NUCB2* rs214101 with multimodality imaging may therefore represent a promising approach for future assessment of tumor aggressiveness and vascular invasion in HCC.

Several limitations of this study should be acknowledged. First, the small numbers of patients in certain genetic subgroups and with positive clinical outcomes, particularly lymph-node metastasis and distant metastasis, may compromise the stability of the estimated associations and increase the risk of overfitting in multivariable logistic regression models. Second, no formal correction for multiple comparisons was applied; therefore, the possibility of false-positive findings cannot be excluded, and the observed associations should be interpreted as exploratory. Third, comprehensive functional validation of the association between NUCB2 genetic variants and HCC progression remains lacking, and NUCB2 and nesfatin-1 expression levels were unavailable in our cohort, precluding evaluation of their relationship with genetic variants and HCC progression. Finally, metabolic factors, including body mass index (BMI) and metabolic dysfunction-associated steatohepatitis (MASH), were not systematically recorded and could not be incorporated into the analyses. Future studies with larger independent cohorts, comprehensive metabolic and expression data, and appropriate functional and mechanistic investigations are warranted to validate these findings and clarify the potential role of the NUCB2/nesfatin-1 pathway in HCC progression.

## 5. Conclusions

In conclusion, our investigation represents the first study to find a connection between variations in the *NUCB2* gene and HCC. Compared with the wild-type GG genotype, the AA genotype at the *NUCB2* SNP rs214101 was associated with an increased risk of distant metastasis and vascular invasion. Furthermore, the GA+AA genotypes at rs214101 were also associated with a greater risk of vascular invasion in HCC patients who smoke. The present results indicate that *NUCB2* SNPs may be associated with distant metastasis and vascular invasion in patients with HCC.

## Figures and Tables

**Table 1:** The distributions of demographical characteristics in 822 normal controls and 411 patients with HCC.

Variable	Controls (*n* = 822)	Patients (*n* = 411)	*p* Value
Age (yrs)			
<60	322 (39.2%)	139 (33.8%)	*p* = 0.067
≥60	500 (60.8%)	272 (66.2%)	
Gender			
Male	606 (73.7%)	288 (70.1%)	*p* = 0.176
Female	216 (26.3%)	123 (29.9%)	
Cigarette smoking			
No	500 (60.8%)	259 (63.0%)	*p* = 0.456
Yes	322 (39.2%)	152 (37.0%)	
Alcohol use			
No	698 (84.9%)	282 (68.6%)	*p* < 0.001 *
Yes	124 (15.1%)	129 (31.4%)	
HBsAg			
Negative		226 (55.0%)	
Positive		185 (45.0%)	
Anti-HCV			
Negative		261 (63.5%)	
Positive		150 (36.5%)	
Stage			
I+II		309 (75.2%)	
III+IV		102 (24.8%)	
Tumor T status			
T1+T2		314 (76.4%)	
T3+T4		97 (23.6%)	
Lymph node status			
N0		402 (97.8%)	
N1+N2+N3		9 (2.2%)	
Metastasis			
M0		387 (94.2%)	
M1		24 (5.8%)	
Vascular invasion			
No		361 (87.8%)	
Yes		50 (12.2%)	
Liver cirrhosis			
Negative		56 (13.6%)	
Positive		355 (86.4%)	

* *p* value < 0.05 was considered statistically significant.

**Table 2:** Genotyping and allele frequency of *NUCB2* single-nucleotide polymorphisms (SNPs) in HCC patients and normal controls.

Variable	Controls (*n* = 822) (%)	Patients (*n* = 411) (%)	AOR (95% CI)	*p* Value
rs1330				
CC	323 (39.3%)	158 (38.4%)	1.000 (reference)	
CT	395 (48.1%)	201 (48.9%)	1.023 (0.787~1.330)	*p* = 0.862
TT	104 (12.6%)	52 (12.7%)	1.029 (0.694~1.527)	*p* = 0.885
CT+TT	499 (60.7%)	253 (61.6%)	1.012 (0.893~1.147)	*p* = 0.848
rs214101				
GG	550 (66.9%)	277 (67.4%)	1.000 (reference)	
GA	234 (28.5%)	119 (29.0%)	1.042 (0.794~1.367)	*p* = 0.768
AA	38 (4.6%)	15 (3.6%)	0.764 (0.407~1.432)	*p* = 0.401
GA+AA	272 (33.1%)	134 (32.6%)	1.001 (0.879~1.139)	*p* = 0.992
rs757081				
CC	331 (40.3%)	160 (38.9%)	1.000 (reference)	
CG	397 (48.3%)	198 (48.2%)	1.043 (0.803~1.355)	*p* = 0.750
GG	94 (11.4%)	53 (12.9%)	1.195 (0.803~1.778)	*p* = 0.381
CG+GG	491 (59.7%)	251 (61.1%)	1.035 (0.914~1.173)	*p* = 0.584
rs10766383				
TT	219 (26.6%)	96 (23.4%)	1.000 (reference)	
TG	409 (49.8%)	227 (55.2%)	1.231 (0.914~1.659)	*p* = 0.171
GG	194 (23.6%)	88 (21.4%)	1.067 (0.747~1.525)	*p* = 0.721
TG+GG	603 (73.4%)	315 (76.6%)	1.086 (0.942~1.252)	*p* = 0.254

Adjusted for the effects of age, gender, cigarette smoking and alcohol use.

**Table 3:** Odds ratios (ORs) and 95% confidence intervals (CIs) for clinical status and *NUCB2* rs214101 genotypic frequencies in 411 patients with HCC.

Variable	*NUCB2* rs214101						
	(*n* = 277)	GA (*n* = 119)	AA (*n* = 15)	GA+AA (*n* = 134)	*p* Value ^a^	*p* Value ^b^	*p* Value ^c^
Clinical Stage							
Stages I/II	208 (75.1%)	91 (76.5%)	10 (66.7%)	101 (75.4%)	0.770	0.465	0.950
Stages III/IV	69 (24.9%)	28 (23.5%)	5 (33.3%)	33 (24.6%)			
Tumor size							
T1+T2	213 (76.9%)	91 (76.5%)	10 (66.7%)	101 (75.4%)	0.927	0.364	0.733
T3+T4	64 (23.1%)	28 (23.5%)	5 (33.3%)	33 (24.6%)			
Lymph node metastasis							
No	270 (97.5%)	118 (99.2%)	14 (93.3%)	132 (98.5%)	0.274	0.339	0.502
Yes	7 (2.5%)	1 (0.8%)	1 (6.7%)	2 (1.5%)			
Distant metastasis							
No	261 (94.2%)	114 (95.8%)	12 (80.0%)	126 (94.0%)	0.522	0.030 ^d^	0.937
Yes	16 (5.8%)	5 (4.2%)	3 (20.0%)	8 (6.0%)			
Vascular invasion							
No	248 (89.5%)	103 (86.6%)	10 (66.7%)	113 (84.3%)	0.392	0.007 ^e^	0.130
Yes	29 (10.5%)	16 (13.4%)	5 (33.3%)	21 (15.7%)			
HBsAg							
Negative	159 (57.4%)	58 (48.7%)	9 (60.0%)	67 (50.0%)	0.112	0.843	0.157
Positive	118 (42.6%)	61 (51.3%)	6 (40.0%)	67 (50.0%)			
Anti-HCV							
Negative	172 (62.1%)	80 (67.2%)	9 (60.0%)	89 (66.4%)	0.330	0.871	0.393
Positive	105 (37.9%)	39 (32.8%)	6 (40.0%)	45 (33.6%)			
Liver cirrhosis							
Negative	39 (14.1%)	15 (12.6%)	2 (13.3%)	17 (12.7%)	0.695	0.935	0.700
Positive	238 (85.9%)	104 (87.4%)	13 (86.7%)	117 (87.3%)			

^a^ indicates the *p*-value for the comparison between GG and GA. ^b^ indicates the *p*-value for the comparison between GG and AA. ^c^ indicates the *p*-value for the comparison between GG and GA+AA. ^d^ OR (95% CI): 4.078 (1.044–15.923). ^e^ OR (95% CI): 4.276 (1.367–13.374). ORs and their 95% CIs were estimated by logistic regression models. *p* < 0.05 was considered statistically significant.

**Table 4:** Odds ratios (ORs) and 95% confidence intervals (CIs) for clinical status and *NUCB2* rs214101 genotypic frequencies in 152 HCC patients who smoke.

Variable	*NUCB2* rs214101						
	GG (*n* = 104)	GA (*n* = 40)	AA (*n* = 8)	GA+AA (*n* = 48)	*p* Value ^a^	*p* Value ^b^	*p* Value ^c^
Clinical Stage							
Stages I/II	76 (73.1%)	29 (72.5%)	5 (62.5%)	34 (70.8%)	0.944	0.519	0.774
Stages III/IV	28 (26.9%)	11 (27.5%)	3 (37.5%)	14 (29.2%)			
Tumor size							
T1+T2	77 (74.0%)	29 (72.5%)	5 (62.5%)	34 (70.8%)	0.851	0.478	0.679
T3+T4	27 (26.0%)	11 (27.5%)	3 (37.5%)	14 (29.2%)			
Lymph node metastasis							
No	101 (97.1%)	40 (100.0%)	7 (87.5%)	47 (97.9%)	0.278	0.158	0.774
Yes	3 (2.9%)	0 (0.0%)	1 (12.5%)	1 (2.1%)			
Distant metastasis							
No	97 (93.3%)	39 (97.5%)	6 (75.0%)	45 (93.8%)	0.321	0.067	0.912
Yes	7 (6.7%)	1 (2.5%)	2 (25.0%)	3 (6.2%)			
Vascular invasion							
No	93 (89.4%)	31 (77.5%)	5 (62.5%)	36 (75.0%)	0.064	0.027 ^d^	0.021 ^e^
Yes	11 (10.6%)	9 (22.5%)	3 (37.5%)	12 (25.0%)			
HBsAg							
Negative	61 (58.7%)	17 (42.5%)	4 (50.0%)	21 (43.8%)	0.081	0.633	0.087
Positive	43 (41.3%)	23 (57.5%)	4 (50.0%)	27 (56.2%)			
Anti-HCV							
Negative	63 (60.6%)	26 (65.0%)	4 (50.0%)	30 (62.5%)	0.625	0.557	0.821
Positive	41 (39.4%)	14 (35.0%)	4 (50.0%)	18 (37.5%)			
Liver cirrhosis							
Negative	15 (14.4%)	4 (10.0%)	1 (12.5%)	5 (10.4%)	0.482	0.881	0.497
Positive	89 (85.6%)	36 (90.0%)	7 (87.5%)	43 (89.6%)			

^a^ indicates the *p*-value for the comparison between GG and GA. ^b^ indicates the *p*-value for the comparison between GG and AA. ^c^ indicates the *p*-value for the comparison between GG and GA+AA. ^d^ OR (95% CI): 5.073 (1.064–24.184). ^e^ OR (95% CI): 2.818 (1.141–6.960). ORs and their 95% CIs were estimated by logistic regression models. *p* < 0.05 was considered statistically significant.

## Data Availability

The original contributions presented in this study are included in the article. Further inquiries can be directed to the corresponding author.
